# To Float or Not to Float: How Interactions between Light and Dissolved Inorganic Carbon Species Determine the Buoyancy of *Stratiotes aloides*

**DOI:** 10.1371/journal.pone.0124026

**Published:** 2015-04-24

**Authors:** Sarah F. Harpenslager, Alfons J. P. Smolders, Ariët A. M. Kieskamp, Jan G. M. Roelofs, Leon P. M. Lamers

**Affiliations:** 1 Department of Aquatic Ecology and Environmental Biology, Institute for Water and Wetland Research, Radboud University, Nijmegen, The Netherlands; 2 B-Ware Research Centre, Nijmegen, The Netherlands; Mediterranean Agronomic Institute at Chania, GREECE

## Abstract

Structural diversity formed by dense, floating *Stratiotes aloides* stands, generates hotspots of biodiversity of flora and fauna in wetlands. However, only part of the populations become emergent and provide this important facilitation. Since it has been hypothesised that its buoyancy depends on the rates of underwater photosynthesis, we investigated the role of dissolved CO_2_ availability and PAR on photosynthesis, biomass production and buoyancy in a controlled greenhouse experiment. Photosynthesis and growth were strongly influenced by both PAR and CO_2_ availability. At low PAR, plants formed less biomass and produced no emergent leaves, even when CO_2_ was abundant. At low CO_2_ levels, *S*. *aloides* switched to HCO_3_^-^ use, resulting in a lower photosynthetic O_2_ production, decreased emergent leaf formation and increased CaCO_3_ precipitation on its leaves, all of which impaired buoyancy. At high PAR, low CO_2_ availability resulted in slower colonisation of the water layer, whereas CO_2_ availability did not influence PAR-limited plants. Our study shows that site conditions, rather than the sole abundance of potentially facilitating species, may strongly determine whether or not they form the structure necessary to act as a facilitator for biodiversity in aquatic environments.

## Introduction

In the natural succession of wetlands, the process of terrestrialisation, the transition from an aquatic to a terrestrial phase, is very important in shaping a structurally diverse habitat and supporting high biodiversity [1, 2]. However, changes in land use and water quality during the 20^th^ century have severely influenced the functioning of wetlands in Western Europe [2, 3] and natural succession of open water to species-rich marshes through the formation of floating sediments or vegetation mats has become rare [1]. Floating stands of emergent aquatic macrophytes, such as *Stratiotes aloides*, can provide structure for a wide variety of flora and fauna species, including the endangered green hawker (*Aeshna viridis*) and black tern (*Chlidonias niger*) [4–6]. Furthermore, biodiversity in these vegetation types is much higher than in similar systems where the species is absent [7, 8].

*S*. *aloides* used to be very common in Europe and North-western Asia until the 1960s but has since declined severely by changes in hydrology, by eutrophication and by ammonium (NH_4_^+^) or sulphide (H_2_S) toxicity [3, 9, 10]. Through their high clonal growth rates, *S*. *aloides* populations can completely fill up surface waters within a few growing seasons under meso- to eutrophic conditions [11]. Such dense vegetation also decreases turbidity of the water layer by preventing re-suspension of sediment particles [12]. Furthermore, the species produces allelopathic substances that reduce algal growth [13]. As a result of these traits, the species is a strong competitor in aquatic systems and has even been reported as a noxious weed in parts of North America and Australia, where it is non-native [14–16].

One of the most characteristic features of *S*. *aloides* is its alternating life cycle with an emergent and submerged life stage [11]. During summer, plants may become buoyant and can form dense floating vegetation mats. In autumn, they sink and remain submerged throughout the winter until they re-emerge in spring. The increased buoyancy of *S*. *aloides* in spring has been speculated to be the result of enhanced underwater photosynthesis [9, 11, 17, 18]. However, field observations and reports from literature clearly show that not all *S*. *aloides* stands follow this alternating life cycle and that populations may remain submerged during summer [17, 19, 20]. Understanding the factors that determine whether a population remains submerged or becomes emergent is important, not only to understand the ecophysiology of floating macrophytes, but also because submerged populations show a different community composition and much lower biodiversity than emergent populations [21] and are unable to reproduce generatively [11, 22].

So far, the mechanism behind buoyancy of *S*. *aloides* has not yet been unravelled under controlled experimental conditions, but as it has been hypothesised to be the direct result of photosynthesis, the absence of floating plants will most probably relate to decreased submerged photosynthetic activity. Reduced photosynthesis may not only be the result of limited nutrient (phosphorus, nitrogen) availability, but also of limited light (PAR; photosynthetically active radiation) conditions, or reduced dissolved inorganic carbon (DIC) availability. Increased turbidity and the presence of humic acids or algae can indeed limit PAR for *S*. *aloides* and thereby its photosynthetic rate [23]. C limitation, on the other hand, may not only occur in softwater lakes, typically showing low DIC concentrations, but also in alkaline waters with increased pH (>8.2) in which DIC is present as bicarbonate (HCO_3_^-^) instead of carbon dioxide (CO_2_) [24]. Aquatic plant species differ considerably in their efficiency to use HCO_3_^-^ as an alternative to CO_2_ [25, 26]. Although *S*. *aloides* is able to use HCO_3_^-^ [27], it may only reach approximately 15% of its maximum net photosynthesis when grown solely on HCO_3_^-^ [17].

To understand the ecophysiological traits and the potential role of *S*. *aloides* as a facilitating species, it is essential to understand the optimal abiotic conditions for floating mat formation. In this study, we therefore tested the interacting effects of PAR and CO_2_ availability on the buoyancy of *S*. *aloides* in a controlled greenhouse experiment. It was hypothesised that plants that experienced either limited PAR or low CO_2_ availability would survive but not become buoyant, since their photosynthetic capacity would be impaired. This process was expected to be even more profound when both CO_2_ and PAR are limiting. Given their facilitating role, research into the regulation of buoyancy of *S*. *aloides* plants is also highly relevant for restoration projects focussing on the natural succession of wetlands and the conservation of endangered species.

## Materials and Methods

### Experimental set-up

In total, 54 *Stratiotes aloides* plants were collected from a privately owned garden pond in the Netherlands, with permission from the owner (51°44’6”N; 6°51’49”E), in autumn 2011 and kept in artificial ponds inside the greenhouse facility of the Radboud University Nijmegen, where the experiment was carried out between April and July 2012. Nine experimental ponds of 2300 L (ø 180 cm; h 90 cm) were each divided into three equally sized compartments by nets to ensure free water movement. One compartment was covered with a double layer of shadow fabric, one with a single layer and one was kept clear of fabric to ensure full light conditions. As a result, three light intensities were created. Taking full light as 100% PAR intensity, shading led to ±40% and ±10% of full PAR intensity for the shaded and double-shaded treatments respectively. Light was mostly natural, but an artificial light regime with 200 μmol m^-2^s^-1^ lamps (Philips, Master SonT, 400W) of 16h light/8h dark was maintained to prevent large fluctuations in the light availability. Average PAR levels at the water surface level reached 130 ± 32, 50 ± 22 and 15 ± 5 μmol m^-2^ s^-1^ (mean ± SEM) for the 100%, 40% and 10% PAR treatments respectively.

Before the addition of the plants, all experimental units received 125 L of underwater peat sediment (see Table 1 for sediment characteristics) originating from a minerotrophic peatland area in the Netherlands (52°18′32″N; 4°45′42″E) and a water layer of 1500 L (60 cm) Nijmegen tap water. Since the experimental basins were dug into the greenhouse floor, water temperature remained quite constant (Median: 23.3°C; Min: 21.1°C; Max: 24.8°C). As a result of the alkaline, bicarbonate (HCO_3_^-^) rich sediments, surface water pH was 7.8 ± 0.1 (mean ± SEM) and CO_2_ concentration was 86 ± 53 μmol L^-1^ (mean ± SEM). Two higher CO_2_ treatments were created by gently bubbling the water layer with pure CO_2_ 4–8 times a day using mass flow controllers (EL-FLOW select F201CV, Bronkhorst, Veenendaal, the Netherlands). These two treatments had concentrations of 228 ± 106 μmol L^-1^ (pH 7.4 ± 0.1; mean ± SEM) and 933 ± 436 μmol L^-1^ (pH 7.0 ± 0.1; mean ± SEM) CO_2_. In the Results and Discussion sections, treatments are called 90, 230 and 930 μmol L^-1^ respectively. During the experiment, the CO_2_ concentrations of the water layer were measured three times a week using an ABB Advance Optima Infrared Gas Analyser (ABB Analytical, Frankfurt, Germany) and treatments remained significantly different throughout the experiment (*P* = 0.018; Table 1).

**Table 1:** Statistical results (*P*- and F-values) of CO_2_, PAR and their interactions on plant parameters and sediment nutrient development during the experiment.

Characteristic	CO_2_		PAR		CO_2_ * PAR	
	*P*	F	*P*	F	*P*	F
**CO**_**2**_ **treatment**	**0.000**	233.082				
**NH**_**4**_^**+**^ **decrease**	**0.005**	5.485	**0.000**	15.028	**0.024**	2.912
**Cover increase**	**0.002**	6.682	**0.000**	40.760	**0.012**	3.386
**Vegetative reproduction**	**0.013**	4.617	**0.000**	79.765	**0.016**	3.265
**Rosette depth**	**0.000**	23.377	**0.000**	37.767	**0.005**	3.883
**CaCO**_**3**_ **accumulation**	**0.001**	8.057	**0.000**	17.285	0.581	0.721
**Final biomass**	0.386	1.005	**0.000**	53.006	0.506	0.861
**No of roots**	**0.018**	5.336	**0.000**	17.160	0.566	0.763
**Final length of roots**	0.712	0.348	**0.001**	11.617	0.068	2.745
**DW/ FW ratio**	0.552	0.615	**0.005**	7.341	0.468	0.931
**Leaf thickness**	0.061	3.401	**0.006**	7.432	0.631	0.658
**Photosynthesis**	**0.003**	8.067	**0.000**	12.911	0.335	1.225
**PAM**	0.668	0.415	**0.002**	9.847	0.152	1.966
**Chlorophyll**	0.499	0.722	**0.012**	5.647	0.288	1.356
**C: N ratio**	0.055	3.447	**0.000**	15.307	0.509	0.856
**C: P ratio**	0.723	0.330	0.163	2.022	0.349	1.194
**N: P ratio**	0.180	1.898	0.242	1.546	0.749	0.481
**C**	**0.006**	6.984	**0.004**	7.937	0.202	1.677
**N**	**0.004**	7.882	**0.000**	22.340	0.093	2.373
**P**	0.764	0.274	**0.008**	6.411	0.740	0.495
**K**	0.893	0.113	0.507	0.706	0.376	1.128
**Fe**	**0.012**	5.815	0.747	0.297	0.389	1.098

Significant *P*-values (*P*<0.05) are indicated in bold.

### Chemical analyses

Water layer and pore water samples were collected every two weeks. Since the water layers of the three compartments of each mesocosm were connected and pH and CO_2_ concentrations showed no differences between the compartments, analyses were performed on one pooled sample from the water layer of each mesocosm. Furthermore, pore water samples of all individual compartments were taken using vacuum syringes attached to ceramic cups (Eijkelkamp Agrisearch Equipment, Giesbeek, The Netherlands) that were fixed at a depth of 10 cm in the sediment. pH was measured with a standard combined glass Ag/AgCl pH electrode (Orion Research, Beverly, CA, USA) connected to a pH meter (Tim800; Radiometer analytical, Lyon, France) and alkalinity by titrating down to pH 4.2 with 0.1 mmol L^-1^ HCl using an auto burette (ABU901, Radiometer, Lyon, France). Concentrations of NO_3_^-^ and NH_4_^+^ were measured colourimetrically on an auto analyser 3 system (Bran&Lubbe, Norderstedt, Germany) using hydrazine sulphate [28] and salicylate [29] respectively. Concentrations of Ca, Fe, K, Mg, total-P and S were analysed by inductively coupled plasma spectrometry (ICP-OES icap 6000; Thermo Fischer scientific, Waltham, MA, USA).

### Plant parameters

While being kept in the greenhouse facility during winter 2011–2012, all plants shed their roots and started forming new ones in April 2012, just before the start of the experiment. Fresh weights, numbers of leaves and offsets, and lengths of the three largest leaves were recorded for each plant before placing two *S*. *aloides* plants (24.6 ± 1.6 g DW; diameter 48 ± 0.5 cm; mean ± SEM) in each compartment. The plants were allowed to grow for 4 months, during which the position of the rosette relative to the water surface (measure of buoyancy), production of emergent leaves, number of offsets and the number of roots penetrating the sediment were recorded regularly. Furthermore, plant mortality during the experiment was recorded and remaining biomass of these dead plants was harvested prematurely. Since it was not possible to record biomass during the experiment without damaging the plants, plant coverage was estimated digitally (Photoshop CS6 for Mac, Measurements Tool; Adobe Systems Incorporated, Mountain View, CA, USA) from pictures that were taken every two weeks, to determine increase in plant cover. After harvest, root lengths were measured and the fresh weights of shoots, roots and offsets were determined separately. Plant material was dried at 70°C for 48h to establish final plant biomass dry weights. These values were corrected for calcium carbonate (CaCO_3_) precipitation.

At the start and at the end of the experiment, the maximum quantum yields of photochemistry (F_v_/F_M_) were measured for all plants using a Pulse Amplified Modulation fluorometer (JUNIOR-PAM, Walz, Effeltrich, Germany). This method provides a relative measure of the reaction centres of photosystem II that are actively involved in photosynthesis. Also, at the start and at the end of the experiment, medium aged leaves were collected for analysis of chlorophyll content, which was extracted and measured on a spectrophotometer (Lambda 25, UV/VIS Spectrometer, PerkinElmer Instruments), according to Lichtenthaler and Wellburn [30].

Photosynthetic rates of medium aged, submerged leaves were measured after 5 weeks of experimental treatments by incubating freshly cut leaves in airtight, water filled flasks. O_2_ production inside the flasks was determined after 0, 2 and 4 hours of incubation using an oxygen electrode (HQ40d multi, HACH, Loveland, Colorado, U.S.A.). We chose to measure after 5 weeks, since this was within the crucial period where all plants were still submerged but were expected to become emergent soon. Measurements were carried out at 195 μmol m^-2^ s^-1^ PAR intensity, created by artificial light (Master SonT, 400W, Philips, The Netherlands). To maintain experimental light conditions, flasks containing leaves from 40% and 10% light treatments were covered with single and double layers of shadow fabric respectively and flasks were filled with water from the corresponding CO_2_ treatments. Measurements were carried out at room temperature (22.5°C).

CaCO_3_ precipitation on leaves was quantified after 25, 50 and 65 days by incubating leaves in airtight bottles completely filled with 0.1 mmol L^-1^ HCl for 24 hours, after which the CO_2_ concentration of the solution was measured using an ABB Advance Optima Infrared Gas Analyser (ABB Analytical, Frankfurt, Germany) and the amount of CaCO_3_ was calculated. Homogenised dried plant material was digested with HNO_3_ (65%) and H_2_O_2_ (30%) using a microwave oven (mls 1200 Mega, Milestone Inc., Sorisole, Italy). Digestates were diluted and analysed for Fe, K and P by inductively coupled plasma spectrometry (ICP-OES icap 6000; Thermo Fischer Scientific, Waltham, MA, U.S.A.). In addition, C and N contents (%) of dried plant material were determined using an elemental analyser (Carlo Erba NA1500, Thermo Fisher Scientific, Waltham, MA, U.S.A.).

### Statistical analyses

Our experimental set-up ensured a full-factorial experiment containing all possible combinations of CO_2_ (90 μmol L^-1^, 230 μmol L^-1^ and 930 μmol L^-1^) and PAR (100%, 40% and 10%) treatments, all of which were replicated 3 times. All replicates consisted of 2 plants in the same compartment.

Normality of residuals and homogeneity of variance were checked using the Shapiro-Wilk Test for Normality and Levene’s Test of Equality of Error Variances, respectively. Non-normal and heteroscedastic data were log transformed or square-rooted before analyses in order to meet the assumptions of parametric tests. Data on final biomass, water chemistry, chemical composition of plant material and photosynthetic parameters were analysed by two-way ANOVAs at the 0.05 confidence limit followed by a Tukey post hoc test. Experimental CO_2_ concentrations, pore water NH_4_^+^ concentrations, plant cover, offset production, buoyancy depth and CaCO_3_ accumulation were analysed over time using linear mixed models. Pairwise comparisons were performed using a Bonferroni adjustment for multiple comparisons to determine significant differences between all possible combinations of CO_2_ or PAR treatments. For all analyses, *P* and F values and interaction effects are presented in Table 1. All statistical tests were carried out using SPSS v21 (IBM Statistics, 2012).

## Results

### Biogeochemistry

Due to the HCO_3_^-^-rich sediments (chemical properties shown in Table 2), characteristic for minerotrophic mires, alkalinity of the water layer in the ponds was high and ranged from 2.4 meq L^-1^ to 3.8 meq L^-1^ (mean: 2.8 meq L^-1^). In general, nutrient concentrations in the water layer were low (Table 2). Pore water NH_4_^+^ concentrations decreased by 17–53% for 10% PAR plants and by 78–99% for plants with 40% or 100% PAR during the experiment (*P*<0.001; data not shown). CO_2_ concentrations of 230 or 930 μmol L^-1^ also resulted in a stronger decrease than when plants were grown with 90 μmol L^-1^ CO_2_ (*P* = 0.004). An interaction effect between PAR and CO_2_ (*P* = 0.024) indicated that the average decrease of 98 ± 1% observed in plants grown at ≥230 μmol L^-1^ CO_2_ and ≥40% PAR (Table 1) was significantly stronger than the 56 ± 13% for those grown at <230 μmol L^-1^ and <40% PAR.

**Table 2:** Chemical composition and characteristics (mean ± SEM) of the water layer, sediment pore water and sediment moisture and organic matter content.

	Water layer	Sediment
Alkalinity (meq L^-1^)	2.85±0.11	9.82 ±0.25
Total Inorganic Carbon (TIC) (mmol L^-1^)	3.57±0.37	11.86±0.42
Ca^2+^ (mmol L^-1^)	3.14±0.14	11.96±0.31
Fe^2+^ (μmol L^-1^)	0.50±0.04	186.83±11.50
K^+^ (mmol L^-1^)	0.22±0.01	0.50±0.02
Mg^2+^ (mmol L^-1^)	0.63±0.02	2.16±0.06
SO_4_^2-^ (mmol L^-1^)	2.48±0.14	9.98±0.28
NH_4_^+^ (μmol L^-1^)*	0.73±0.01	79.52±12.88
NO_3_^-^ (μmol L^-1^)	74.41±9.52	2.06±0.80
Total P (μmol L^-1^)	0.68±0.17	21.95±1.14
Moisture content (%)		50±7
Organic matter content (%)		20±6

* For results of statistical analyses on the decline of NH_4_^+^ concentrations in the pore water during the experiment, see Table 1. Please note the different units.

### Plant growth and photosynthesis

**Fig 1:**
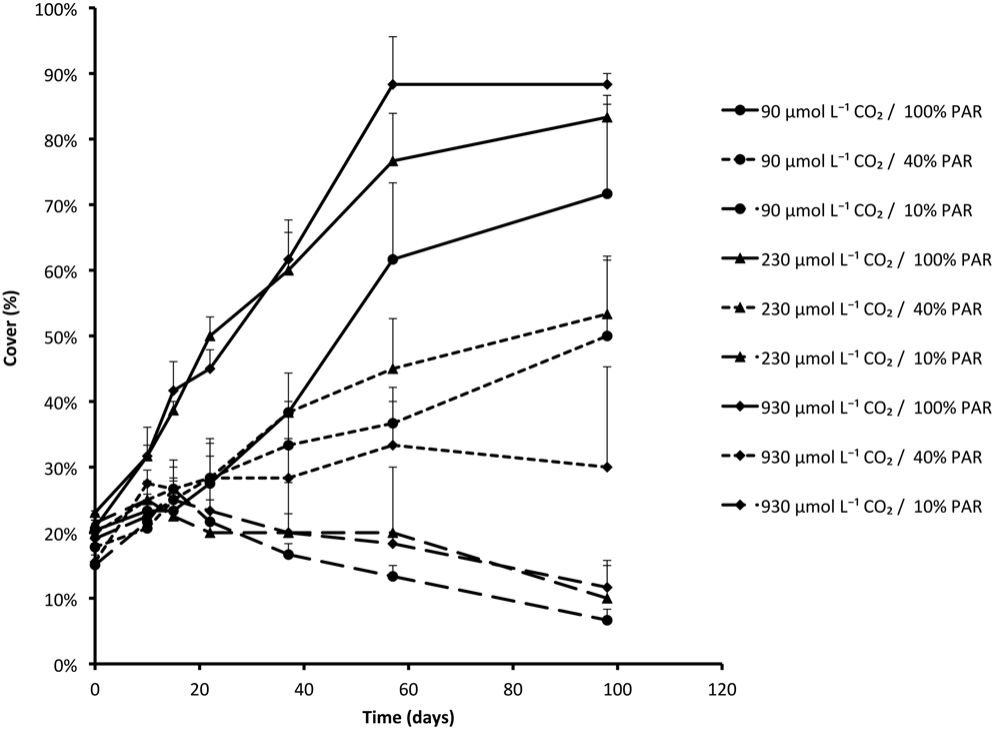
Increase in cover of the water layer by *Stratiotes aloides* (mean + SEM) subjected to different PAR levels and CO_2_ availability. Low PAR significantly reduced final cover (*P*<0.001), whereas CO_2_ limitation resulted in slower colonisation rates (*P* = 0.001). Results of statistical tests are presented in Table 1.

**Fig 2:**
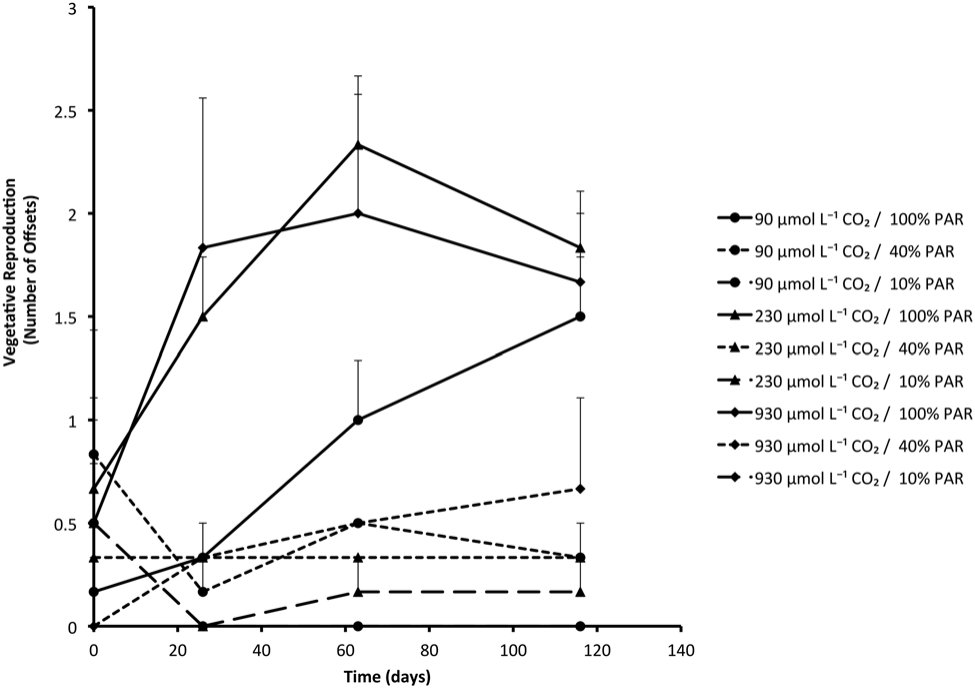
Production of offsets through vegetative reproduction in *Stratiotes aloides* (mean + SEM) subjected to different PAR levels and CO_2_ availabilities. Low PAR significantly reduced the ability to produce offsets (*P*<0.001), whereas CO_2_ limitation resulted in slower production rates (*P* = 0.021). Results of statistical tests are presented in Table 1.

PAR was the most important factor determining final biomass of *Stratiotes aloides* (Table 3). While the biomass of PAR-limited plants decreased during the experiment due to the production of thinner leaves (*P* = 0.006; Table 3) and shedding of large leaves, plants grown under 100% PAR had produced approximately three times their initial weight after four months of experimental treatments. Furthermore, these plants covered the water layer faster and more completely (*P*<0.001; Fig 1), and had a higher vegetative reproduction than PAR-limited plants (*P*<0.001; Table 1; Fig 2). Plants grown under 100% PAR produced 1.7 ± 0.2 offsets per plant, whereas plants grown under 40% or 10% PAR conditions produced only 0.4 ± 0.2 and 0.05 ± 0.05 offsets respectively.

**Table 3:** Plant growth characteristics (mean ± SEM) of *Stratiotes aloides* after 4 months of different PAR and CO_2_ treatments.

		100% PAR			40% PAR			10% PAR		
Characteristic	Unit	90 μmol L^-1^ CO_2_	230 μmol L^-1^ CO_2_	930 μmol L^-1^ CO_2_	90 μmol L^-1^ CO_2_	230 μmol L^-1^ CO_2_	930 μmol L^-1^ CO_2_	90 μmol L^-1^ CO_2_	230 μmol L^-1^ CO_2_	930 μmol L^-1^ CO_2_
Final Plant Biomass	g DW	129.5±30.7 ^C^	168.4±13.6 ^C^	128.7±19.1 ^C^	53±11.3 ^B^	63.7±12.3 ^B^	49.7±10.5 ^B^	19.9±2.8 ^A^	16.5±9.4 ^A^	27.3±2.9 ^A^
Chlorophyl a+b	mg Chl g DW^-1^	0.79±0.04 ^A^	0.68±0.18 ^A^	0.65±0.09 ^A^	1.24±0.26 ^B^	0.95±0.15 ^B^	0.82±0.08 ^B^	0.93±0.12 ^B^	1.14±0.14 ^B^	1.09±0.02 ^B^
Photosynthetic Yield (PAM)	F_V_/F_M_	0.70±0.01 ^A^	0.64±0.04 ^A^	0.72±0.02 ^A^	0.72±0.01 ^A^	0.76±0.01 ^A,B^	0.73±0.03 ^A,B^	0.75±0.01 ^B^	0.77±0.01 ^B^	0.78±0.01 ^B^
Final number of roots		3.3±1.6 ^B^	5.8±2.6 ^B^	8.7±2.0 ^B^	2.0±1.2 ^A,B^	3.3±0.9 ^A,B^	5.3±0.8 ^A,B^	1.7±1.4 ^A^	0.5±0.3 ^A^	1.3±0.7 ^A^
Final length of roots	cm	88.2±4.0 ^B^	84.3±9.2 ^B^	94.3±4.4 ^B^	95.0±1.4 ^B^	81.3±12.1 ^B^	74.3±1.3 ^B^	36.5±0.4 ^A^	69.3±2.2 ^A^	65.7±14.8 ^A^
DW/FW ratio leaves		0.25±0.06 ^B^	0.24±0.03 ^B^	0.20±0.02 ^B^	0.17±0.02 ^A^	0.13±0.03 ^A^	0.16±0.003 ^A^	0.14±0.01 ^A^	0.13±0.01 ^A^	0.14±0.01 ^A^
Leaf thickness	mm	0.53±0.12 ^B^	0.61±0.01 ^B^	0.56±0.06 ^B^	0.44±0.07 ^A,B^	0.53±0.06 ^A,B^	0.40±0.02 ^A,B^	0.23±0.01 ^A^	0.47±0.01 ^A^	0.39±0.03 ^A^
Emergent leaf formation	weeks	15	9	6	-	-	-	-	-	-

Significant differences among PAR treatments are indicated by different capital letters (A, B, C). See Table 1 for *P* and F values.

All plants started producing roots approximately 1 week before the start of the experiment and the plants were all firmly rooted in the sediment after 3 weeks. Plants grown at 100% PAR produced more roots (*P*<0.001; Table 1; Table 3) than those from the other light treatments, while plants from the lowest PAR treatment produced the shortest roots (*P* = 0.001; Table 1; Table 3). From this 10% PAR treatment, however, a substantial part of the plants died (±40%, compared to ±20% and ±5% of the plants in the 40% and 100% PAR treatments respectively), which started with the dying off of the roots. A sufficient number of plants survived, however, for statistical analyses of plant parameters. The total length of the roots from all treatments, apart from the 10% PAR and 90 μmol L^-1^ CO_2_ treatment, measured more than 60 cm and thus easily reached the sediment of the experimental basins (Table 3).

CO_2_-limited plants initially showed much lower growth rates than plants with ample CO_2_. This was illustrated by both lower increase in plant cover (*P* = 0.001; Table 1; Fig 1) and slower production of offsets (*P* = 0.021; Table 1; Fig 2). Plants from 90 μmol L^-1^ CO_2_ treatments grown with full PAR eventually reached similar coverage and number of offsets, but took much longer to reach these values than plants from 930 μmol L^-1^ CO_2_ availability.

Increase in plant cover (*P* = 0.012) and vegetative production (*P* = 0.016) were both also significantly influenced by interaction effects of CO_2_ and PAR, since one factor enhanced the effects of the other. When plants received both high CO_2_ concentrations and high PAR, the effects were stronger than the separate effects. Similarly, plants grown at the lowest CO_2_ concentration and the lowest PAR did significantly worse than plants limited by only one of these factors.

**Fig 3:**
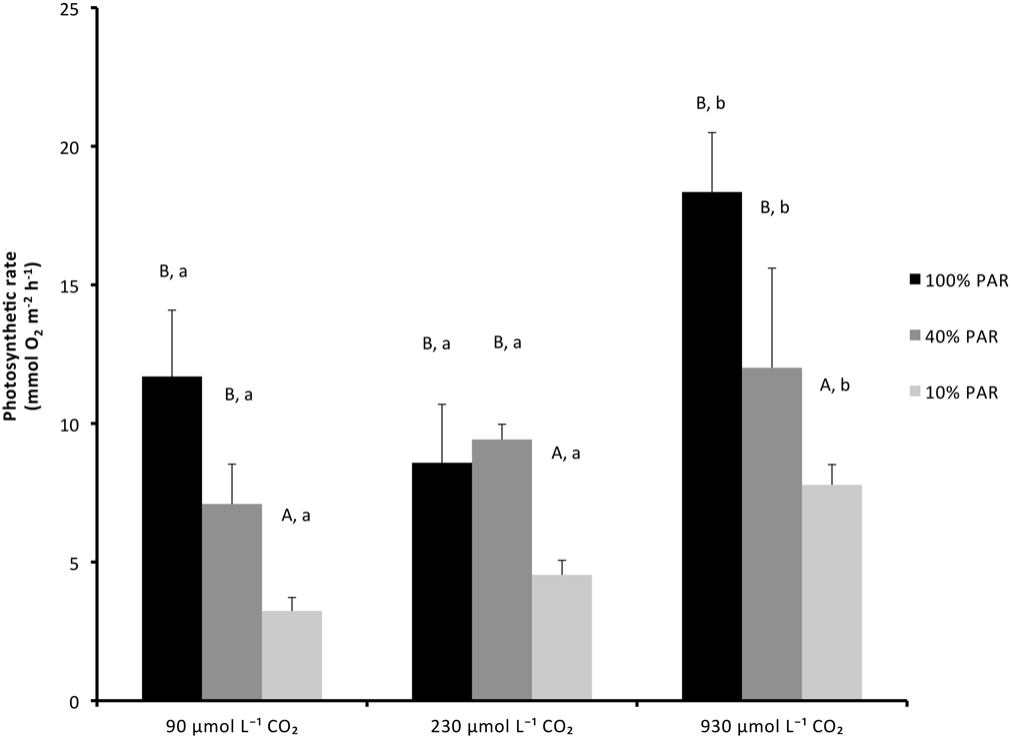
Photosynthetic oxygen production (mean + SEM) in *Stratiotes aloides* under different PAR and CO_2_ availabilities. Significant differences between different PAR and CO_2_ treatments are indicated with capital and lower cased letters, respectively (P<0.01). Additional results of statistical tests are presented in Table 1.

The photosynthetic rates of the submerged plants were strongly influenced by both PAR (*P*<0.001; Table 1; Fig 3) and CO_2_ availability (*P* = 0.003; Table 1; Fig 3). Reduced PAR led to a reduction of approximately 50% of O_2_ production in plants from the 10% PAR treatment compared to those grown at full PAR. Furthermore, compared to plants grown at 930 μmol L^-1^, the photosynthetic rates of plants from the 90 μmol L^-1^ and 230 μmol L^-1^ CO_2_ treatments were reduced by 55% and 40% respectively.

Maximum quantum yield values showed that all of the remaining plants had an active photosystem at the end of the experiment. These values were significantly higher for plants grown at 40% and 10% PAR than for those grown in the 100% PAR treatment (*P* = 0.002; Table 1; Table 3). Furthermore, plants that were grown under darker conditions contained significantly more chlorophyll a and b than those grown in full light (*P* = 0.009; Table 1; Table 3).

After harvest, the C: N ratios of the plants grown at 40% and 10% PAR were lower than those of the plants grown in full PAR (*P* = 0.010; Table 1; Table 4). Furthermore, plants grown at 930 μmol L^-1^ CO_2_ had significantly higher N contents than those of lower CO_2_ availability, which also led to lower C: N ratios. C: P and N: P ratios did not differ among treatments (Table 4). Plants also showed lower Fe contents (*P* = 0.012; Table 1; Table 4) when CO_2_ concentrations were 230 μmol L^-1^ or lower, while K contents did not differ among treatments, with all plants containing around 630±50 mmol K kg DW^-1^ (Table 4; mean ± SEM).

**Table 4:** Plant chemical composition (mean ± SEM) of *Stratiotes aloides* after 4 months of different PAR and CO_2_ treatments.

		100% PAR			40% PAR			10% PAR		
Characteristic	Unit	90 μmol L^-1^ CO_2_	230 μmol L^-1^ CO_2_	930 μmol L^-1^ CO_2_	90 μmol L^-1^ CO_2_	230 μmol L^-1^ CO_2_	930 μmol L^-1^ CO_2_	90 μmol L^-1^ CO_2_	230 μmol L^-1^ CO_2_	930 μmol L^-1^ CO_2_
C	mol kg DW^-1^	^a,b^ 21.77±1.76 ^A^	^a^ 22.53±1.92 ^A^	^b^ 24.06±2.51 ^A^	^a,b^ 23.10±1.47^A^	^a^ 21.63±1.24 ^A^	^b^ 24.47±1.12 ^A^	^a,b^ 25.85±0.24 ^B^	^a^ 24.24±2.07 ^B^	^b^ 25.44±2.44 ^B^
N	mmol kg DW^-1^	^a^ 788±269 ^A^	^a^ 947±299 ^A^	^b^ 1066±390 ^A^	^a^ 909±366 ^B^	^a^ 708±107 ^B^	^b^ 914±39 ^B^	^a^ 1204±394 ^C^	^a^ 958±295 ^C^	^b^ 1338±311 ^C^
P	mmol kg DW^-1^	36.09±2.82 ^A^	25.54±7.13 ^A^	36.42±4.71 ^A^	38.65±2.98 ^A, B^	38.28±3.76 ^A. B^	41.06±9.64 ^A, B^	47.66±3.80 ^B^	57.39±14.75 ^B^	55.34±11.13 ^B^
K	mmol kg DW^-1^	675±54	412±113	567±123	819±186	618±145	566±166	520±99	814±167	743±250
Fe	mmol kg DW^-1^	^a^ 3.50±1.39	^a^ 4.34±0.11	^b^ 9.27±3.05	^a^ 2.34±0.37	^a^ 5.41±0.85	^b^ 11.29±1.91	^a^ 7.01±2.62	^a^ 6.11±2.44	^b^ 7.84±2.86
C: N	g g^-1^	^a,b^ 33.70±4.90 ^B^	^b^ 42.79±0.65 ^B^	^a^ 28.52±3.29 ^B^	^a,b^ 25.62±5.45 ^A^	^b^ 27.19±3.90 ^A^	^a^ 16.90±1.93 ^A^	^a,b^ 19.85±7.58 ^A^	^b^ 14.80±1.17 ^A^	^a^ 13.80±0.25 ^A^
C: P	g g^-1^	929±96	1453±252	1052±126	898±55	850±81	1152±126	862±58	669±45	874±135
N: P	g g^-1^	30.06±5.55	33.92±11.59	38.78±6.46	37.75±2.31	33.47±1.83	72.58±4.53	54.50±6.89	44.78±2.90	64.02±7.38

Significant differences among PAR treatments are indicated on the right by different capital letters (A, B, C) while significant differences among CO_2_ treatments are indicated on the left by different lower case letters (a, b, c). See Table 1 for *P* and F values. Please note a different unit for C-content (mol kg DW^-1^).

### Buoyancy and calcification

**Fig 4:**
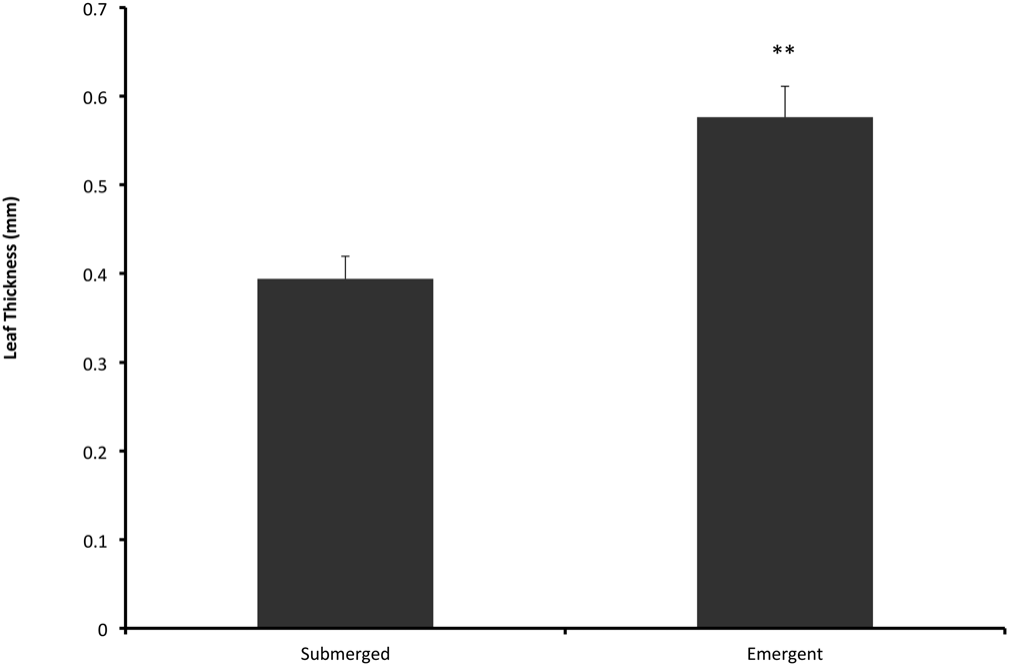
Thickness of submerged and emergent leaves (mean + SEM) formed by *Stratiotes aloides* at the end of the four-month experimental treatments. Submerged leaves were mainly produced by PAR-limited plants and plants from 90 μmol L^-1^ treatments, whereas emergent leaves were only produced by plants grown at 100% PAR. Emergent leaves were significantly thicker than submerged leaves (*P* = 0.002). Average values of leaf thickness per treatment are presented in Table 3, with statistical details in Table 1.

Under full PAR, formation of emergent leaves started in the 930 μmol L^-1^ CO_2_ treatments after approximately 6 weeks (Table 3). Under limited CO_2_ availability, formation of emergent leaves was delayed by another 3 and 9 weeks for the 230 μmol L^-1^ and 90 μmol L^-1^ CO_2_ treatments respectively. When PAR was limited, plants did not form emergent leaves. Submerged leaves were significantly thinner than emergent leaves (*P* = 0.002; Table 1; Fig 4).

**Fig 5:**
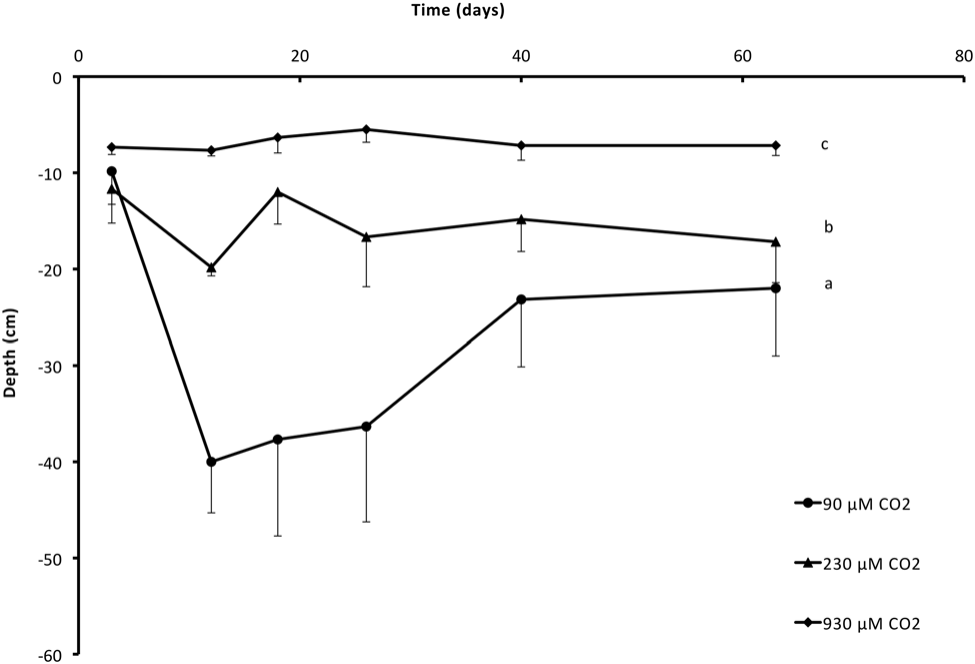
Depths of the rosettes (mean and SEM) of *Stratiotes aloides*, grown at different CO_2_ availabilities and 100% PAR. Plants from 90 μmol L^-1^ CO_2_ treatments sank within two weeks, after which plants remained significantly lower in the water layer than those grown at limited 230 and 930 μmol L^-1^ (*P*<0.001), as indicated by different letters. Additional results of statistical tests are presented in Table 1.

Still, even in the absence of emergent leaves, PAR-limited plants were often observed to float just below the water surface and buoyancy depth of the rosette did not differ between light regimes for plants grown at 230 μmol L^-1^ and 930 μmol L^-1^ CO_2_. However, at 100% light, plants that received the lowest amount of CO_2_ were lying lower in the water layer than plants with 230 or 930 μmol L^-1^ during the entire experimental period (CO_2_*PAR effect; *P* = 0.005; Table 1; Fig 5). Furthermore, plants from 230 μmol L^-1^ CO_2_ treatments initially also stayed lower in the water layer (*P* = 0.017; Table 1; Fig 5) than plants treated with 930 μmol L^-1^ CO_2_.

**Fig 6:**
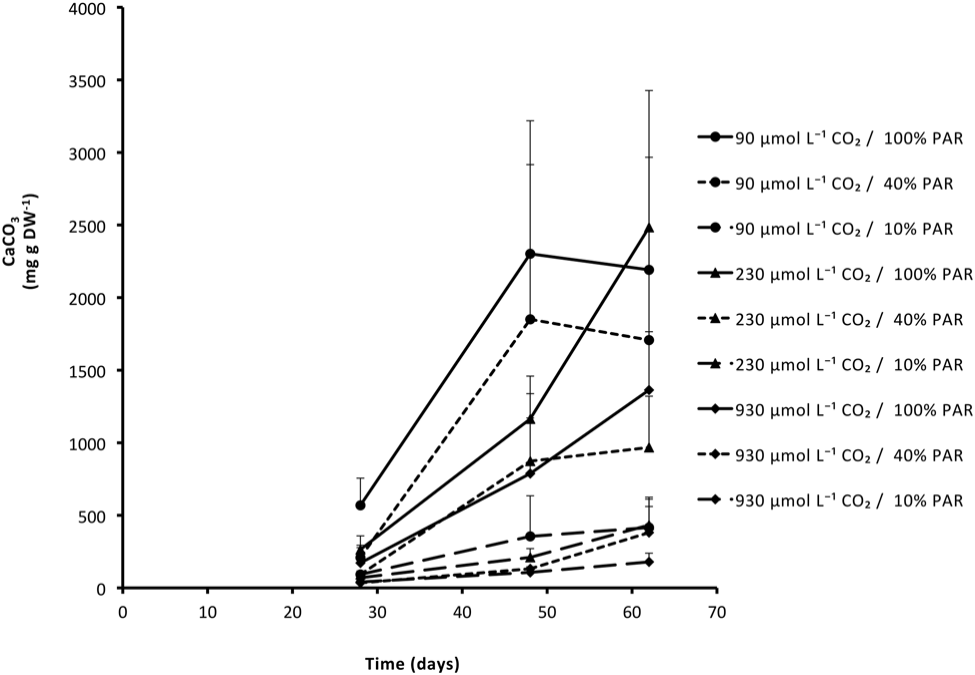
Accumulation of CaCO_3_ (mean + SEM) on leaves of *Stratiotes aloides* under different PAR and CO_2_ availabilities. CaCO_3_ was measured from the moment the precipitation became visible (3–4 weeks after start of treatments). Low PAR significantly lowered CaCO_3_ accumulation (*P* = 0.002), while limited availability of CO_2_ significantly increased the amount of CaCO_3_ on the leaves (*P* = 0.016). Additional results of statistical tests are presented in Table 1.

Observations of submerged leaves showed significant accumulation of, what proved to be, CaCO_3_ on the upper side of the leaves. While this accumulation was most apparent on plants from the 90 μmol L^-1^ CO_2_ and 100% PAR treatment, eventually all plants showed some CaCO_3_ precipitation on their leaves. This calcification of *S*. *aloides* leaves first became visible after approximately 3–4 weeks. Both high PAR (*P* = 0.002; Table 1) and low CO_2_ availability (*P* = 0.016; Table 1) significantly increased the amount of CaCO_3_ that accumulated on the leaves (Fig 6). The increase in CaCO_3_ was highest during the first month of the experiment and stabilised after that to values of around 40 ± 7 g CaCO_3_ m^-2^ (data not shown). Accumulation of CaCO_3_ during the experiment led to an increase in leaf density of 5.7 ± 0.9% in full PAR, whereas those in 40% and 10% PAR increased 3.2±1.0% and 1.8±0.4% respectively (data not shown).

## Discussion

### Buoyancy and photosynthesis

**Fig 7:**
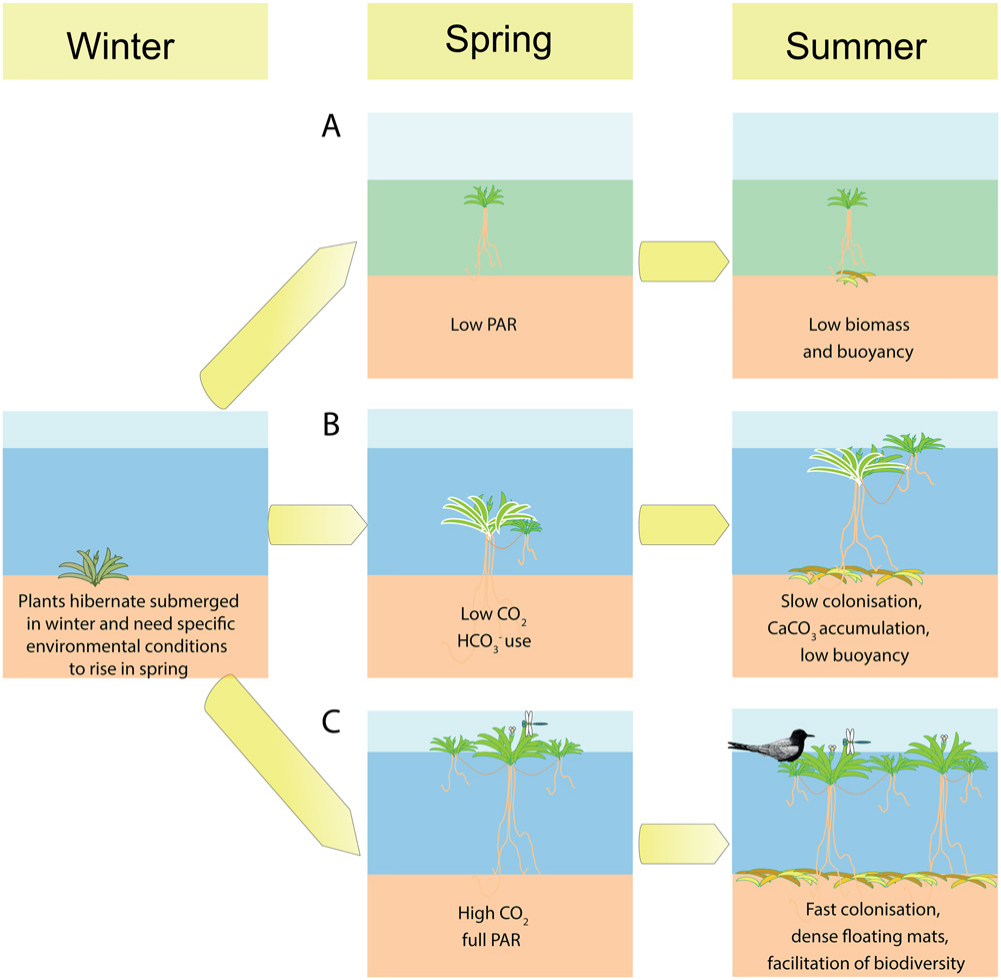
Schematic overview of the effects of limited PAR or CO_2_ availability in spring on the growth rate and buoyancy of *Stratiotes aloides*. When PAR is limited, plants produce less biomass and do not form emergent leaves. Under limited CO_2_ conditions, plants use HCO_3_^-^ for photosynthesis (when available), which is less efficient and causes lower growth rates, slower formation of emergent leaves and reduced buoyancy. Therefore, only plants receiving ample light and CO_2_ can become buoyant and reach the required growth rate necessary to fulfil their role as a facilitator for biodiversity in wetlands.

We show here that both PAR and the availability of dissolved CO_2_ in the water layer strongly influence photosynthetic rates, buoyancy and formation of emergent leaves for *Stratiotes aloides*. A synthesis of the most important effects of limited CO_2_ or PAR on these plants is presented in Fig 7.

Emergent leaves did not appear at all when PAR was limiting, whereas their formation was delayed by 3–9 weeks under CO_2_ limited conditions. All plants were firmly rooted in the sediment and produced roots that were long enough for the plants to become buoyant. Root growth, therefore, was not a constraint on reaching the water surface. PAR-limited plants, however, never became emergent but they floated just below the water surface (Fig 7). We observed that these plants show several adaptations to low light availability, including thinner leaves, a higher chlorophyll content and a higher efficiency of photosystem II. Still, the photosynthetic rate is too low to produce emergent leaves and form a dense floating mat under PAR-limiting conditions and several plants died or shed their roots during the experiment, resulting, on average, in shorter roots. We observed that PAR limited plants, which happened to have shed all of their roots still did not become buoyant. This further indicates that plants were not physically retained under water due to a limited root length. In the field, low PAR may well be caused by blooms of algae or cyanobacteria, high concentrations of humic acids or turbidity by particulate organic matter, all of which occur frequently in peatlands where *S*. *aloides* occurs [2].

With full PAR, however, CO_2_ limitation can still cause reduced buoyancy (Fig 7). This can be explained by the lower photosynthetic rates of these plants and their subsequently delayed formation of emergent leaves. Even though both light and DIC availability were high enough for photosynthesis in this treatment, the lower photosynthetic rate indicates C-limitation for these plants. Emergent leaves are thicker and have a larger volume of gas-filled intercellular spaces (aerenchyma) than submerged leaves, and are able to provide buoyancy to the plants [11, 18, 31]. The construction costs, in terms of energy and carbon, involved in the formation of the structurally more complex, thicker emergent leaves are much higher [32] and this C allocation is only possible if photosynthesis is sufficiently high. Even though *S*. *aloides* is capable of using HCO_3_^-^ as an alternative C-source [17, 27], photosynthetic rates are lower due to the higher costs associated with HCO_3_^-^ use [33]. This would explain why, under natural conditions, this species strongly prefers systems with an organic sediment, where high microbial respiration rates ensure high CO_2_ concentrations in the water layer [9].

Furthermore, as we have shown, HCO_3_^-^-based photosynthesis induces accumulation of CaCO_3_ on plant leaves. Calcification of aquatic plants is a common phenomenon occurring in species that use HCO_3_^-^ [34]. When aquatic plants take up HCO_3_^-^, it is converted to CO_2_ and OH^-^. While CO_2_ is used for photosynthesis, OH^-^ is excreted on the upper side of the leaf, thereby locally increasing the pH of the boundary layer and causing precipitation of CaCO_3_ [34, 35]. Accumulation of CaCO_3_ on *S*. *aloides* leaves has been discovered in the last century, when Montesantos [36] argued that CaCO_3_ accumulation could explain the natural sinking of the species in autumn. Even though we could not find a direct link between CaCO_3_ accumulation and limited buoyancy, we established that HCO_3_^-^ use increases the density of plant tissue in *S*. *aloides*.

### Biomass production and plant nutrition

Buoyancy is the key factor determining the function of *S*. *aloides* as a facilitator for wetland biodiversity. However, to create dense vegetation mats that can facilitate other species, the growth rate and production of sufficient new biomass are equally important (Fig 7). Plants grown at low PAR produced significantly lower amounts of new biomass than plants with high PAR. Plants were generally smaller and produced fewer offsets than those grown at full PAR availability (Fig 7). Since both PAR and CO_2_ influence photosynthesis, it is not surprising that the effects of CO_2_ or PAR limitation interact with each other. While plants grown at full PAR performed best when grown at high CO_2_ concentrations, PAR-limited plant propagation did not respond to variations in CO_2_ availability. Plants that were limited in CO_2_, but received sufficient PAR for photosynthesis, used HCO_3_^-^ as an alternative C-source. This not only reduced buoyancy, but also resulted in slower growth and reproductive rates (Fig 7). In wetlands, slower growth rates will easily result in plants being outcompeted by more efficient HCO_3_^-^ using species, including both plants and algae.

While growth rates were lower under CO_2_ limited conditions, there were no significant differences between the final biomasses of the lowest and highest CO_2_ treatments. This can most likely be explained by space restrictions in plants grown at high CO_2_, since these plants already covered over 90% of the basin after 60 days. At this point, space would limit recruitment of new offsets due to self-shading and other intra-specific competition factors.

Even though NH_4_^+^ availability dropped in sediments of plants with high CO_2_ and high PAR, N limitation can be ruled out, since N: P ratio’s of all plants were higher than 30 g g^-1^ which suggests that P, rather than N, was limiting biomass production in all treatments [37]. Furthermore, even though tissue of CO_2_ limited plants had lower Fe contents than those grown with ample CO_2_, all concentrations were within the range of healthy *S*. *aloides* vegetation, as was K, and Fe or K limitation can thus be ruled out [38, 39].

### Habitat quality and facilitator function

We have shown that one of the most important characteristics making *Stratiotes aloides* a facilitator species for biodiversity, buoyancy, can only be achieved when the plants are able to reach sufficiently high underwater photosynthetic rates in spring. High photosynthesis enables high colonisation rates and the formation of thicker leaves with intercellular spaces that can become filled with gas, most likely oxygen produced by photosynthesis. The resulting lower specific weight of the plants makes them emerge to the surface. This process is strongly linked to PAR availability and to the species of C, rather than the total DIC concentration, in the aquatic environment. Therefore, contrary to many other facilitators, the potential of this widespread aquatic macrophyte to facilitate other species appears to depend on the prevalent environmental conditions, rather than the mere abundance of the species.

## References

[1] Verhoeven JTA, Bobbink R (2001) Plant diversity of fen landscapes in The Netherlands In: Gopal B., editor editors. Biodiversity in wetlands: assessment, function and conservation. Leiden: Blackhuys Publishers pp. 65–87.

[2] Lamers LPM, Smolders AJP, Roelofs JGM (2002) The restoration of fens in The Netherlands. Hydrobiologia 478: 107–130.

[3] Roelofs JGM (1991) Inlet of alkaline river water into peaty lowlands: effects on water quality and *Stratiotes aloides* L. stands. Aquatic Botany 39: 267–293.

[4] Suhonen J, Suutari E, Kaunisto KM, Krams I (2013) Patch area of macrophyte *Stratiotes aloides* as a critical resource for declining dragonfly *Aeshna viridis* . Journal of Insect Conservation 17: 393–398.

[5] Rantala MJ, Ilmonen J, Koskimaki J, Suhonen J, Tynkkynen K (2004) The macrophyte, *Stratiotes aloides*, protects larvae of dragonfly *Aeshna viridis* against fish predation. Aquatic Ecology 38: 77–82.

[6] Van der Winden J, Beintema AJ, Heemskerk L (2004) Habitat-related Black Tern (*Chlidonias niger*) breeding success in The Netherlands. Ardea 92: 53–61.

[7] Strzałek M, Koperski P (2009) The *Stratiotes aloides* L. stand as a habitat in oxbow lake Bużysko. Aquatic Botany 90: 1–6.

[8] Sugier P, Lorens B, Chmiel S, Turczyński M (2010) The influence of *Ceratophyllum demersum* L. and *Stratiotes aloides* L. on richness and diversity of aquatic vegetation in the lakes of mid-eastern Poland. Hydrobiologia 656: 43–53.

[9] Smolders AJP, Lamers LPM, den Hartog C, Roelofs JGM (2003) Mechanisms involved in the decline of *Stratiotes aloides* L. in The Netherlands: sulphate as a key variable. Hydrobiologia 506: 603–610.

[10] Smolders AJP, Roelofs JGM (1996) The roles of internal iron hydroxide precipitation, sulphide toxicity and oxidizing ability in the survival of *Stratiotes aloides* roots at different iron concentrations in sediment pore water. New Phytologist 133: 253–260. 29681078 10.1111/j.1469-8137.1996.tb01892.x

[11] Cook CDK, Urmi-König K (1983) A revision of the genus *Stratiotes* (Hydrocharitaceae). Aquatic Botany 16: 213–249.

[12] Madsen JD, Chambers PA, James WF, Koch EW, Westlake DF (2001) The interaction between water movement, sediment dynamics and submersed macrophytes. Hydrobiologia 444: 71–84.

[13] Mulderij G, Smolders AJP, Donk E (2006) Allelopathic effect of the aquatic macrophyte, *Stratiotes aloides*, on natural phytoplankton. Freshwater Biology 51: 554–561.

[14] NRM-SA (2011) State Alert Weed: Stratiotes aloides. In: N. R. M. G. o. S. Australia, editor editors.

[15] MNR (2013) Ontario’s Invasive Aquatic Plant List. In: O. M. o. N. Resources, editor editors. ontarioca/invasivespecies.

[16] NRCS (2014) State noxious weed lists for 46 states. In: U. S. D. o. A. N. R. C. Service, editor editors. https://plants.usda.gov/java/invasiveOne.

[17] Nielsen LT, Borum J (2008) Why the free floating macrophyte *Stratiotes aloides* mainly grows in highly CO_2_-supersaturated waters. Aquatic Botany 89: 379–384.

[18] Sculthorpe CD (1967) The biology of aquatic vascular plants. London, UK: Edward Arnold 610 p.

[19] Erixon G (1979) Population ecology of a *Stratiotes aloides* L. stand in a riverside lagoon in N Sweden. Hydrobiologia 67: 215–221.

[20] Renman G (1989) Life histories of 2 clonal populations of *Stratiotes aloides* L. Hydrobiologia 185: 211–222.

[21] Higler LWG (1977) Macrofauna-cenoses on *Stratiotes* plants in Dutch broads. Rijksinstituut voor Natuurbeheer.

[22] Toma C (2006) Distribution and comparison of two morphological forms of water soldier (Stratiotes aloides L.): a case study on Lake Słosineckie Wielkie (Northwest Poland). Biodiversity: Research and Conservation 3–4: 251–257.

[23] Bloemendaal FHJL, Roelofs JGM (1988) Waterplanten en Waterkwaliteit. Utrecht: Stichting Uitgeverij van de Koninklijke Natuurhistorische Vereniging.

[24] Stumm W, Morgan JJ (1996) Aquatic Chemistry In: Schnoor J. L. and Zehnder A., editors. Current Contents/Agriculture Biology & Environmental Sciences. New York, U.S.A.: John Whiley & Sons pp. 157–163. doi: 10.7717/peerj.328

[25] Allen ED, Spence DHN (1981) The differential ability of aquatic plants to utilize the inorganic carbon supply in fresh waters. New Phytologist 87: 269–283.

[26] Madsen TV, Maberly SC (1991) Diurnal-variation in light and carbon limitation of photosynthesis by 2 species of submerged freshwater macrophyte with a differential ability to use bicarbonate. Freshwater Biology 26: 175–187.

[27] Prins HBA, De Guia MB (1986) Carbon source of the water soldier, *Stratiotes aloides* L. Aquatic Botany 26: 225–234.

[28] Kamphake LJ, Hannah SA, Cohen JM (1967) Automated analysis for nitrate by hydrazine reduction. Water Research 1: 205–&.

[29] Grasshof K, Johannse H (1972) New sensitive and direct method for automatic determination of ammonia in seawater. Journal Du Conseil 34: 516–521.

[30] Lichtenthaler HK, Wellburn AR (1983) Determinations of total carotenoids and chlorophylls a and b of leaf extracts in different solvents. Biochemical Society Transactions 11: 519–520.

[31] Efremov AN, Sviridenko BF (2012) Seasonal and spatial dynamics of *Stratiotes aloides* (Hydrocharitaceae) plants. Botanica Serbica 36: 59–62.

[32] Milla R, Reich PB (2007) The scaling of leaf area and mass: the cost of light interception increases with leaf size. Proceedings of the Royal Society B-Biological Sciences 274: 2109–2114. 17591590 10.1098/rspb.2007.0417 PMC2706187

[33] Madsen TV, Sand-Jensen K (1991) Photosynthetic carbon assimilation in aquatic macrophytes. Aquatic Botany 41: 5–40.

[34] Borowitzka MA (1984) Calcification in aquatic plants. Plant Cell and Environment 7: 457–466.

[35] Prins HBA, Snel JFH, Zanstra PE, Helder RJ (1982) The mechanism of bicarbonate assimilation by the polar leaves of *Potamogeton* and *Elodea*—CO_2_ concentrations at the leaf surface. Plant Cell and Environment 5: 207–214.

[36] Montesantos N (1913) Morphologische und biologische Untersuchungen über einige Hydrocharideen. Flora 105: 1–32.

[37] Güsewell S (2004) N: P ratios in terrestrial plants: variation and functional significance. New Phytologist 164: 243–266. 10.1111/j.1469-8137.2004.01192.x 33873556

[38] Smolders A, Roelofs JGM (1993) Sulfate mediated iron limitation and eutrophication in aquatic ecosystems. Aquatic Botany 46: 247–253.

[39] Marschner H (1995) Mineral Nutrition in Higher Plants. London: Academic Press Limited 889 p.

